# Strengthening Dental Materiovigilance: A Review of International Reporting Frameworks

**DOI:** 10.1016/j.identj.2025.100943

**Published:** 2025-08-07

**Authors:** Shalya Anand, Ömer Faruk Sönmez

**Affiliations:** a Center for Adaptive Rationality, Max Planck Institute for Human Development, Berlin, Germany; b Subject Area Dental Devices and Materials, Materiovigilance Program of India, New Delhi, India; c Oral Health Literacy Division, International Health Literacy Association, St Louis, MO, USA; d School of Medicine and Population Health, University of Sheffield, Sheffield, UK

**Keywords:** Materiovigilance, Dental devices, Adverse event reporting, Patient safety, Regulatory frameworks

## Abstract

Dental and medical devices are fundamental to oral healthcare delivery; however, their growing utilization and increasing variety of products due to the material innovation, introduces potential risks of adverse events. Despite the existence of regulatory frameworks, adverse events are underreported and the awareness among dental professionals remains low. This review presents the first comparative analysis in the literature of international materiovigilance systems for dental devices and examines how harmonized adverse event reporting could improve their global safety, efficacy, and reliability. A narrative review was undertaken, drawing upon peer-reviewed literature and regulatory documents. Comparative analyses were performed across systems in the United States, European Union, United Kingdom, Australia, and India, to explore diversity in regulatory maturity, geographic representation, and influence on global dental device markets. Key areas of focus included regulatory classifications, mandatory reporting requirements, post-market surveillance strategies, and integration into clinical practice. Findings from international frameworks such as the European Union’s Medical Device Regulation (MDR), the U.S. FDA’s Medical Device Reporting system, the UK’s MHRA reporting mechanisms, Australia’s TGA Incident Reporting and Investigation Scheme (IRIS), and India’s Materiovigilance Programme (MvPI), indicate that standardized reporting mechanisms significantly enhance adverse event detection and enable timely interventions. However, discrepancies in regulatory standards, reporting protocols, and stakeholder engagement practices continue to impede the development of a cohesive global materiovigilance framework for dental devices. Effective materiovigilance in dentistry necessitates coordinated international efforts involving regulators, manufacturers, healthcare professionals, and patients. Establishing harmonized systems is crucial to minimizing risks, increasing reporting of adverse events associated with dental devices. Solutions, such as, strengthening reporting infrastructure, promoting stakeholder education, and achieving cross-national regulatory convergence are imperative for advancing dental device safety and efficacy. This review underscores the critical importance of materiovigilance in ensuring the safe use of dental devices. It offers clinicians and regulatory authorities a global overview of strategies to strengthen post-market surveillance and emphasizes the urgent need for standardized adverse event reporting systems for dental materials and devices to protect patient and user safety. Global organizations e.g. World Health Organization (WHO) and the World Dental Federation (FDI) could play a pivotal role in this context, going forwards.

## Introduction

Dental and medical devices are essential components of healthcare systems, significantly contributing to the diagnosis, treatment, and prevention of various diseases. In dentistry, a diverse range of devices ranging from implants and endodontic files to orthodontic brackets, dental chairs, and dental unit water line systems are routinely used in clinical care. While these tools enhance efficiency and patient outcomes, they are not without risk. The U.S. Food and Drug Administration (FDA) defines adverse events as undesirable experiences associated with the use of medical products, including dental devices.^1^ Unlike pharmaceuticals, these devices typically do not directly interact chemically or metabolically with the human body. Nevertheless, their safe use demands continuous vigilance and systematic monitoring to prevent harm and ensure optimal performance.^2^

Patient safety remains a foundational element of quality healthcare delivery.^3^ Yet, globally, preventable adverse events and medical errors continue to present significant threats to health outcomes. Acknowledging this challenge, the Seventy-Second World Health Assembly (2019) adopted Resolution WHA 72.6 on global action for patient safety, which led to the development of the Global Patient Safety Action Plan 2021-2030**.** This plan, endorsed by the Seventy-Fourth World Health Assembly in 2021, envisions "a world in which no 1 is harmed in health care, and every patient receives safe and respectful care, every time, everywhere."^3^ The Global Patient Safety Action Plan provides a policy roadmap for reducing preventable harm through strategic actions at multiple levels from healthcare institutions to national governments. It calls for the alignment of safety standards, the establishment of national safety action plans, and the strengthening of reporting systems and regulatory oversight.^3^

In this context, materiovigilance defined as the active process of detecting, assessing, reporting, and preventing adverse events related to medical devices emerges as a key component of post-marketing surveillance (PMS).^1^^,^^2^^,^^4^, ^5^, ^6^, ^7^, ^8^, ^9^, ^10^, ^11^, ^12^, ^13^, ^14^, ^15^, ^16^, ^17^, ^18^, ^19^, ^20^, ^21^, ^22^, ^23^, ^24^, ^25^, ^26^, ^27^, ^28^, ^29^, ^30^, ^31^, ^32^, ^33^, ^34^, ^35^, ^36^, ^37^ While regulatory approval through conformity assessment allows a device to enter the market, it is the post-market phase that often reveals unanticipated risks encountered in real-world settings. This is especially relevant in dentistry, where certain device failures or malfunctions may not become apparent during initial trials but can cause harm during routine use over a period.^4^

Materiovigilance in dentistry refers to the systematic monitoring, assessment, and reporting of adverse events associated with dental devices. These events encompass device malfunctions, breakages, allergic reactions, infections, and other unforeseen complications that may arise during clinical use.^1^^,^^2^^,^^4^^,^^8^^,^^23^ Multiple dental devices including toothbrushes, cranio-maxillofacial distraction systems, orthodontic components, impression materials, preformed arch wires, milled crown materials, implant instruments, and porcelain glaze sprays have faced global recalls due to safety risks. Counterfeit, non-CE marked devices sold online further endanger users.^6^ These adverse events highlight inconsistent regulatory enforcement and the urgent need for harmonized global standards to ensure patient safety across dental care systems.

Current materiovigilance in dentistry varies globally. Some countries rely on manufacturers and regulators for post-market surveillance, while others involve healthcare providers and patients in reporting. However, under-reporting and limited awareness remain challenges, highlighting the need for improved collaboration and standardized practices. Despite the essential nature of materiovigilance and patient safety, the dental sector remains underserved in the broader discourse on device safety.

This review provides the first comparative analysis in the literature of international materiovigilance frameworks for dentistry. Five regulatory frameworks from US, EU, UK, Australia, and India were selected due to their diverse regulatory maturity, geographic representation, and influence on global dental device markets. Together, they provide a balanced overview of established and evolving materiovigilance systems. Although the analysis does not capture all regional variations, particularly from low-resource settings, it highlights gaps in adverse event reporting, regulatory harmonization, and stakeholder engagement. The review calls for unified, patient- and user-centered approaches, with proactive stakeholder engagement, to ensure the long-term safety and effectiveness of dental devices worldwide.

## Dental devices included under materiovigilance in dentistry

A "medical device" refers to the instrument, appliance, apparatus, material, or article, also including the necessary software, intended by manufacturer for use in humans for: diagnosis, monitoring, treatment, prevention, or the alleviation of diseases, the compensation for an injury and handicap, the investigation, replacements, or modification of the anatomy or physiological process, controls of conception.^8^ A variety of dental devices, among others, are categorized and presented in Table 1.^9^

**Table 1:** Common dental devices and categories.

Category	Devices
Diagnostic Instruments	Mouth mirrors, straight probes, double-ended probes, periodontal probes, tweezers, instrument trays, kidney trays, endo locking tweezers, Williams probes, CPITN probes, Nabers probes, pocket markers, cotton holders
Dental Instruments & Equipment	Dental operating chairs, air compressors, suction pumps, handpieces, sterilization devices, dental drills, saliva ejectors, scalers, curettes, burnishers, pluggers, retractors, dental lasers, dental torque wrenches, dental unit water delivery systems
Restorative & Filling Materials	Dental fillings, composite resins, dental cements, amalgam fillings, bonding agents, dental impression materials, dental liners and bases, dental bonding systems
Dental Implants	Titanium or zirconia dental implants, abutments, implant-supported prosthetics, bone graft materials, membrane barriers
Orthodontic Appliances	Braces, aligners, retainers, space maintainers, orthodontic bands, orthodontic wires, orthodontic brackets, orthodontic pliers
Prosthetic Devices	Crowns, bridges, removable partial dentures, complete dentures, overdentures, dental veneers, inlays, onlays
Mouthguards & Oral Devices	Sports mouthguards, night guards, mandibular advancement devices (MAD), tongue-retaining devices, speech prostheses, bruxism splints
Dental Imaging Devices	X-ray machines, RVG (Radiovisiography), OPG (Orthopantomogram), intraoral cameras, 3D imaging systems, cone-beam computed tomography (CBCT) scanners
Endodontic Equipment	Apex locators, endodontic explorers, endodontic files and reamers, gutta-percha points, obturation systems, pulp testers
Periodontal Instruments	Scalers, curettes, periodontal probes, ultrasonic scalers, periodontal knives, surgical elevators
Surgical Instruments	Elevators, forceps, surgical handpieces, bone chisels, surgical curettes, suturing instruments, retractors
Laboratory Equipment	Dental articulators, facebows, dental lathes, model trimmers, vacuum mixers, casting machines, porcelain furnaces
General Medical Devices Used in Dental Settings	IV supplies, thermometers, EKG supplies, laparoscopic equipment, needles, syringes, autoclaves, sterilization pouches, patient management software

## Materiovigilance in international regulatory frameworks

### United States

In the United States, the Food and Drug Administration (FDA) is responsible for pre-market evaluation of medical and dental devices through its approval pathways and post-market surveillance, to monitor device performance and safety after commercialization. Once a device is available on the market, manufacturers, importers, and healthcare facilities are required to report any adverse events, malfunctions, or product issues through the Medical Device Reporting system. The FDA may also require post market surveillance studies under Section 522 of the Federal Food, Drug, and Cosmetic Act, which mandates manufacturers to collect additional safety and effectiveness data for certain high-risk devices.^10^ To ensure traceability, the FDA mandates medical device tracking that allows devices to be located throughout distribution, from manufacturing to the end-user, for select Class II and Class III devices, particularly implantable and life-sustaining products. The Unique Device Identification (UDI) system on their labels, has been implemented to improve post-market surveillance. This system enhances traceability, error reduction, and regulatory compliance by allowing regulators, manufacturers, and healthcare providers to monitor device usage more effectively.^11^

### Australia

In Australia, the Therapeutic Goods Administration (TGA) mandates that sponsors (manufacturers and importers) report adverse events related to medical devices through the Incident Reporting and Investigation Scheme (IRIS) as a condition of regulatory approval.^12^ While healthcare professionals and consumers are encouraged but not required to report device-related incidents, their input plays a crucial role in identifying safety concerns. Reports are submitted via the Medical Device Incident Reporting (MDIR) system, allowing the TGA to assess risks, investigate trends, and take necessary regulatory actions to enhance post-market surveillance and patient safety.^13^

### European Union

The EU has transitioned from the Medical Device Directive (MDD) to the Medical Device Regulation (MDR) (EU 2017/745). The MDR introduced stronger post-market requirements, including real-world performance monitoring and enhanced risk management. It also emphasizes stricter safety and performance standards that manufacturers must meet to obtain CE marking prior to market access. Notified Bodies play a critical role in assessing higher-risk devices, while Competent Authorities (CAs) oversee regulatory enforcement. While the shift aims to foster innovation and patient safety, extended transition periods have been granted to prevent market disruptions.^14^ By balancing technological advancement with regulatory stringency, the EU framework ensures that safety remains a top priority.^15^

MDR (EU 2017/745) mandates that manufacturers report serious incidents and Field Safety Corrective Actions (FSCAs) to National Competent Authorities (NCAs). Reporting timelines vary: serious public health threats must be reported within 2 days, incidents causing death or serious deterioration in health within 10 days, and other serious incidents within 15 days. Manufacturers submit a Manufacturer’s Incident Report (MIR), conduct investigations, and provide follow-up reports to NCAs.^16^ The European Database on Medical Devices (EUDAMED), an IT framework, centralizes vigilance data and provides a live picture of the medical devices through its lifecycle, for the public and healthcare professionals, enhancing coordination between different member states in the EU. This ensures timely risk management and regulatory oversight to maintain patient safety and device reliability.^16^

### United Kingdom

 Following Brexit, the United Kingdom established its own regulatory framework for medical devices, UK Medical Device Regulations 2002 (UK Medical Device Regulations 2002), derived from previous EU directives. To place a medical device on the Great Britain market, manufacturers must obtain the UK Conformity Assessed (UKCA) marking, which replaces the EU's CE marking. The Medicines and Healthcare products Regulatory Agency (MHRA) functions as a standalone regulator, ensuring the safety and efficacy of medical devices in the UK.^17^ Manufacturers are mandated to report such incidents through the Manufacturer's On-line Reporting Environment (MORE) system. Healthcare professionals and the public can report issues via the Yellow Card Scheme.^18^ MHRA classifies medical devices based on their intended purpose and associated risk levels. The primary categories include general medical devices, active implantable medical devices (AIMDs), and in vitro diagnostic (IVD) devices. Table 2 outlines these classifications along with their associated regulatory requirements.^19^

**Table 2:** MHRA classification system based on risk levels adopted in UK.

Class	Risk level & conformity pathway	Examples
Class I	Low risk; manufacturer self-declaration	Dental handheld mirror, Reusable dental osteotome (sgs.com)
Class Is	Low risk, sterile; Approved Body for sterility	Sterile examination gloves; surgical mirror in sterile pack
Class Im	Low risk, measuring; Approved Body for measurement	Measuring periodontal probe, Intraoral blood pressure or sensor device
Class IIa	Low-medium risk; Approved Body conformity	Orthodontic wires, Dental filling materials, Dental wound dressings (non-animal source)
Class IIb	Medium-high risk; Approved Body conformity	Dental implants and abutments
Class III	High risk; Approved Body conformity	Periodontal/bone membranes (e.g. bovine collagen)

### India

India has made significant strides in medical device safety in recent years, particularly with the implementation of the Medical Devices Rules 2017, which came into effect on January 1, 2018. These regulations establish a structured framework for the classification, approval, and post-market surveillance of medical devices, including dental products.^20^

The Materiovigilance Programme of India (MvPI), launched by the Ministry of Health and Family Welfare, operates under the coordination of the Indian Pharmacopoeia Commission (IPC). The program aims to establish a comprehensive database of adverse events related to medical and dental devices, monitor and assess their safety, and communicate risks to stakeholders.^21^ Additionally, the MvPI provides evidence-based recommendations to the Central Drugs Standard Control Organization (CDSCO), India's primary regulatory authority for medical devices.^22^ The program's key objectives include establishing a nationwide patient safety monitoring system for medical devices, conducting risk-benefit analyses to ensure the safe use of medical and dental devices, generating evidence-based safety data to support regulatory decision-making, strengthening reporting mechanisms to enhance transparency and encourage active participation from healthcare professionals and manufacturers and collaborating with national and international health organizations to promote best practices in materiovigilance.^23^

Under Medical Device Rules 2017, dental devices in India are classified based on risk levels, aligning with global regulatory standards seen in Table 3.^24^

**Table 3:** Dental device risk classification.

Risk Level	Examples of Dental Devices
Class A (Low Risk)	Dental scissors, reusable dental excavators, manual and electric toothbrushes, impression materials, examination gloves
Class B (Low-Moderate Risk)	Alginate and composite impression materials, bonding agents, polymer dental crowns, curing lights
Class C (Moderate-High Risk)	Dental implants, bone graft materials, X-ray machines, denture adhesives
Class D (High Risk)	TMJ implants, surgical lasers, resorbable bone cements

A synthesized comparison of the 5 frameworks that lists key parameters across the regions are summarized in Table 4.

**Table 4:** Materiovigilance frameworks in Dentistry across 5 settings.

Parameter	United States	European Union	United Kingdom	Australia	India
Regulatory Authority	Food and Drug Administration (FDA)	National Competent Authorities (NCAs), Notified Bodies	Medicines and Healthcare products Regulatory Agency (MHRA)	Therapeutic Goods Administration (TGA)	Central Drugs Standard Control Organization (CDSCO), Indian Pharmacopoeia Commission
Adverse Event Reporting	Mandatory for manufacturers, importers, and healthcare facilities via Medical Device Reporting system	Mandatory for manufacturers via MIR; patients and professionals encouraged	Mandatory for manufacturers via MORE; voluntary via Yellow Card Scheme	Mandatory for sponsors via IRIS; voluntary for others	Voluntary via Materiovigilance Programme of India (MvPI)
Post-Market Surveillance Tools	MDR system, UDI, Section 522 studies	EUDAMED database, FSCAs, vigilance timelines (2-15 days)	UKCA marking, Yellow Card Scheme	MDIR system, incident trend monitoring	MvPI database, risk-benefit analysis, national and international collaborations
Device Classification System	Class I, II, III based on risk	Class I, IIa, IIb, III (per MDR 2017/745)	Class I, Is, Im, IIa, IIb, III (based on risk and function)	Class I (low risk) to Class III (high risk)	Class A (low risk) to Class D (high risk) for dental devices
Digital Tools/ Databases	Unique Device Identification (UDI)	EUDAMED	Yellow Card, MORE	MDIR, IRIS	MvPI online reporting portal
Public/ Professional Engagement	Healthcare professionals required to report; public engagement limited	Professionals encouraged; reports feed into EU-wide vigilance	Professionals/public encouraged to report; open Yellow Card access	Healthcare professionals and consumers encouraged	Awareness-building for HCPs; limited patient involvement

## Strengthening materiovigilance in dentistry: reporting and risk management for dental devices

Materiovigilance in dentistry involves the systematic monitoring, assessment, and reporting of adverse events such as device malfunctions, breakages, allergic reactions, infections, and other unforeseen complications that may arise during its use.^23^^,^^25^ All suspected medical device-associated adverse events (MDAEs) regardless of severity, frequency, or established causality should be reported to ensure comprehensive safety surveillance.^26^

Active participation from dental healthcare professionals in reporting MDAEs is crucial.^27^ Such reporting enhances risk awareness and contributes to overall patient safety. Notably, reports are treated with confidentiality, and patient identities are protected. Serious reports undergo rigorous evaluation, considering factors such as timing, accuracy, and potential contributing elements to ascertain the device's involvement. These assessments facilitate the identification of safety signals and inform regulatory actions, including safety alerts, product recalls, updates to user manuals, and the development of training materials. Ultimately, materiovigilance data bolster benefit-risk analyses and support targeted educational initiatives to promote safer dental device usage across healthcare systems.^28^

Adverse events related to dental devices can be reported by dental and other healthcare professionals, as well as patients and consumers. Reporting centers encompass public and private clinics, hospitals, and research institutions. Events can be submitted using customized paper-based or digital forms.^27^ To validate and assess MDAEs effectively, stakeholders must provide comprehensive information. The establishment of toll-free helplines, can encourage submissions from both healthcare professionals and the public, and facilitate user-friendly reporting.^29^

The comprehensive reporting form should include key details as shown in Table 5 ^26^

**Table 5:** Suspected adverse event reporting requirements ranging from minimum information to root cause analysis.

Type Of information	Details
General information	Date of the report, type of report (initial/follow up/final/trend)
Reporter details	Type of reporter (manufacturer/importer/healthcare professionals and others) and reporter contact information
Device category	Device type (therapeutic/diagnostic/preventive/others), invasive/non-invasive, single use/reusable, sterile/nonsterile and others
Device details	License number, batch/model, manufacturing date, expiry date, and others
Event description	Event date, date of implant, serious/non serious, description of the event and others
Patient information	Patient initial, age, gender, weight, relevant medical history, patient outcomes (recovered/not recovered), and others

## Challenges in advancing materiovigilance in dentistry

Implementing materiovigilance programs in dentistry faces challenges similar to those long recognized in pharmacovigilance, particularly in fostering active participation from both practitioners and patients. Reporting adverse events is often seen as a cumbersome task, and many stakeholders remain unclear about when, how, and why to report. The absence of mandatory reporting regulations further hinders the integration of materiovigilance into daily clinical routines.^30^ Negative perceptions among some practitioners who may equate reporting with admitting clinical fault also acts as a barrier, discouraging transparency and open communication.

A significant hurdle is the shortage of trained personnel and the lack of robust reporting infrastructure., 1 potential solution could involve regulatory bodies requiring that all dental devices include package inserts and a Summary of Product Characteristics (SPC), thereby improving awareness and standardizing reporting processes.^31^

Building a resilient materiovigilance framework is essential to ensure dental devices remain safe, affordable, and accessible. In the European Union, the shift from the Medical Device Directive (MDD) to the MDR has notably raised the standards for post-market surveillance. Although manufacturers retain primary responsibility for monitoring device safety, the MDR imposes stricter oversight through conformity assessment bodies and notified authorities. As highlighted by Mohn and Zehnder (2023), the MDR’s robust framework demands comprehensive clinical evaluations, ongoing post-market surveillance (PMS), and the implementation of Unique Device Identification (UDI), collectively reinforcing patient safety across the device lifecycle.^32^ It requires detailed documentation, risk reclassification, and continuous performance monitoring. These measures enhance device traceability and empower clinical decision-making. As platforms like EUDAMED become increasingly accessible to dental professionals, the benefits of greater transparency and harmonized reporting are expected to grow. Although concerns about administrative burdens, particularly for small and medium-sized enterprises, have been raised, the MDR’s emphasis on safety and consistency offers hope for significantly minimizing adverse events.^33^

Technological and data management limitations present another formidable barrier to effective materiovigilance. Many health systems and regulatory agencies continue to rely on paper-based or fragmented digital reporting mechanisms. This reliance hampers timely detection of safety signals. To overcome this, the development of validated tools for signal detection and causality assessment is critical.^15^ The integration of automated reporting systems, AI-driven signal detection, and real-time analytics could revolutionize materiovigilance practices. However, deploying such technologies will demand substantial investment, clear regulatory pathways, and careful change management.^34^

As dentistry embraces digital workflows, 3D-printed implants, and bioactive materials, ensuring patient safety amid rapid innovation becomes increasingly complex. Existing post-market surveillance frameworks may not be sufficiently agile to address the unique challenges posed by these evolving technologies. Therefore, adapting materiovigilance systems to support innovation while safeguarding public health is a pressing global priority.

## Recommendations for strengthening materiovigilance

To strengthen materiovigilance in dentistry, a comprehensive, multi-stakeholder approach is essential. Globally harmonized guidelines, standardized reporting protocols, and international regulatory collaboration are crucial to ensure consistency and effectiveness. Raising awareness through structured educational initiatives such as workshops, seminars, and practical training is key, alongside integrating materiovigilance into dental curricula and clinical protocols to foster early professional engagement. Accessible digital reporting platforms and centralized databases can streamline adverse event monitoring, while regular audits and feedback mechanisms will enhance accountability. Public awareness campaigns can further empower patients to report issues. Collectively, these measures will build a proactive safety culture, reduce risk, and improve device reliability in oral healthcare.

## Global harmonization and the future of materiovigilance in dentistry

The advancement of materiovigilance in dentistry necessitates a globally coordinated approach, engaging regulatory bodies, manufacturers, healthcare professionals, and patients. Disparities in regulatory frameworks, reporting methodologies, and levels of stakeholder engagement continue to obstruct the development of a harmonized system for dental device safety monitoring.^35^

However, use of Artificial Intelligence (AI) tools can enhance materiovigilance in dentistry by enabling automated detection of adverse events by analyzing large volumes of electronic dental records, clinical notes and patient feedback, analyzing clinical data using natural language processing, and predicting device failures. It can support smart reporting, clinical decision-making, and global data harmonization, ultimately improving safety, early risk identification, and regulatory responsiveness across dental healthcare systems.^36^^,^^37^

International organizations, notably the Global Harmonization Task Force (GHTF), have long advocated for standardized adverse event reporting and have issued guidelines aimed at unifying regulatory processes, expediting reviews, and improving patient safety across jurisdictions.^38^ Continued commitment toward the development of standardized reporting systems and the fostering of cross-border regulatory collaboration is therefore imperative to enhance the detection, management, and prevention of device-related risks on a global scale.

Despite growing international recognition of the importance of materiovigilance in dentistry, achieving global harmonization remains constrained by significant systemic and operational challenges. A key barrier is the lack of regulatory alignment across jurisdictions, with substantial variation in national legislation, approval pathways, and post-market surveillance requirements. This regulatory heterogeneity impedes the development of standardized reporting systems and effective data-sharing mechanisms. Furthermore, disparities in technical capacity, infrastructure, and human resources—particularly in low- and middle-income countries—limit the establishment of robust and sustainable materiovigilance systems. These limitations contribute to persistent underreporting and delays in adverse event identification and response. Additionally, the absence of strong stakeholder incentives and institutional accountability mechanisms continues to hinder active participation from healthcare professionals, manufacturers, and patients.^39^ While the pursuit of a harmonized global framework is both essential and timely, it must be grounded in pragmatic, context-sensitive approaches that address local constraints, bridge capacity gaps, and foster sustained political and institutional commitment.

## Conclusion

The Materiovigilance Programme for dentistry is a vital initiative designed to reinforce post-market surveillance (PMS) of dental devices through structured adverse event reporting, rigorous data analysis, and systematic risk assessment. However, challenges such as underreporting, limited awareness, and procedural complexities underscore the need for stronger stakeholder engagement, more robust regulatory frameworks, and the integration of digital reporting systems to enhance efficiency and transparency. Globally, many countries encounter similar barriers in establishing effective materiovigilance systems, particularly for dental devices and materials. Although regions such as the European Union, United States, United Kingdom, India and Australia have implemented comprehensive PMS regulations and centralized reporting databases, significant disparities remain in dental device vigilance across countries.

To bridge these gaps, ongoing investment in professional education, regulatory reforms, and technological innovation is essential. To address the fragmented landscape of materiovigilance in dentistry, it is imperative that both governmental bodies and non-governmental organizations take an active, coordinating role in developing a unified, dental-specific vigilance framework. Organizations such as WHO, the Global Harmonization Task Force (GHTF), the World Federation of Public Health Associations (WFPHA), and the World Dental Federation (FDI) could play a pivotal role in this context. In particular, the FDI with its connectivity across 200 national member dental associations and specialist groups in some 130 countries should pay heed to the urgent necessity of formulating an international materiovigilance framework for the dental devices and materials. Strengthening international collaboration can contribute to the shared goal towards, "a world in which no 1 is harmed in health care, and every patient receives safe and respectful care, every time, everywhere."

## Author contributions

**Shalya Anand** was responsible for the conception, design, literature review, analysis, drafting, and finalization of the manuscript. **Omer Faruk Sonmez** reviewed the manuscript and contributed insights on the contextual relevance of European and UK frameworks. All authors have read and approved the final manuscript.

## Funding source

None

## Declaration of generative AI and AI-assisted technologies in the writing process

Generative AI tools (e.g., ChatGPT by OpenAI) were used solely to assist with language editing and grammar refinement. The author(s) reviewed and edited the content as needed and took full responsibility for the publication's final version.

## Declarations of competing interests

Dr. Shalya Anand is the Subject Area Expert (Dental Devices and Materials) at the Materiovigilance Program of India, New Delhi.
